# GATA binding protein 4 promotes the expression and transcription of hepatitis B virus by facilitating hepatocyte nuclear factor 4 alpha in vitro

**DOI:** 10.1186/s12985-021-01668-z

**Published:** 2021-09-28

**Authors:** Xiaoqin Lv, Xia Xiang, Yue Wu, Yang Liu, Ruqing Xu, Qin Xiang, Guoqi Lai

**Affiliations:** 1 Laboratory Animal Center of Chongqing Medical University, No. 1, Yixueyuan Road, Yuzhong District, Chongqing, 400016 China; 2 The Second Affiliated Hospital of Chongqing Medical University, Chongqing, 40010 China; 3 LuXian No. 2 High School, Sichuan, 646100 China; 4 Molecular Oncology and Epigenetics Laboratory of the First Affiliated Hospital of Chongqing Medical University, Chongqing, 400016 China

**Keywords:** Hepatitis B virus, GATA binding protein 4, Hepatocyte nuclear factor 4 alpha, Hepatocellular carcinoma, HBV transcription

## Abstract

**Background:**

GATA binding protein 4 (GATA4) has been reported as a potential target of gene therapy for hepatocellular carcinoma (HCC). It is well known that the main cause of HCC is the chronic infection of hepatitis B virus (HBV). However, whether the effect of GATA4 on HBV has not yet been reported.

**Methods:**

In this study, the regulation of GATA4 on HBV was analyzed in vitro. In turn, the effect of HBV on GATA4 was also observed in vitro, in vivo, and clinical HCC patients. Subsequently, we analyzed whether the effect of GATA4 on HBV was related to hepatocyte nuclear factor 4 alpha (HNF4α) in vitro.

**Results:**

The results showed that GATA4 significantly promoted the secretion of HBV surface antigen (HBsAg) and HBV e antigen in the cell culture medium, improved the replication of HBV genomic DNA, and increased the level of HBV 3.5 kb pre-genomic RNA and HBV total RNA (*P* < 0.05). Moreover, it was showed that HBV had no significant effect on GATA4 in vitro and in vivo (*P* > 0.05). At the same time, GATA4 expression was decreased in 78.9% (15/19) of HCC patients regardless of the HBV and HBsAg status. Among them, there were 76.9% (10/13) in HBV-associated patients with HCC (HBV-HCC), and 83.3% (5/6) in non-HBV-HCC patients. In addition, the expression of HNF4α was also up-regulated or down-regulated accordingly when stimulating or interfering with the expression of GATA4. Furthermore, stimulating the expression of HNF4α could only alleviate the HBsAg level and HBV transcription levels, but had no significant effect on GATA4.

**Conclusions:**

In summary, this study found that GATA4 has a positive effect on HBV, and the potential pathway may be related to another transcription factor HNF4α that regulates HBV.

## Introduction

It is reported that the global incidence and mortality of hepatocellular carcinoma (HCC) in 2018 are 4.7% and 8.2% respectively, which is the sixth most common cancer and the fourth leading cause of death for cancer [1]. Treatment methods for HCC include surgical resection, radiotherapy, and chemotherapy, each of with its shortcomings [2]. Gene therapy is considered to be a potential treatment for effectively suppressing HCC with the in-depth research on tumor suppressor genes [3]. GATA binding protein 4 (GATA4), one of the members of the GATA transcription family, is a key transcription factor that regulates cell differentiation and tissue development [4, 5]. Studies have reported that GATA4 can inhibit the development of a variety of cancers [6–9]. Recently, GATA4 has also been found to be an important tumor suppressor gene for HCC [10, 11]. Previously, we found that GATA4 induces mesenchymal-to-epithelial transition and cellular senescence through the NF-κB pathway in hepatocellular carcinoma. GATA4 plays a crucial tumor-suppressive role in HCC [12].

HCC is a primary liver cancer, and its main pathogenic factors include alcoholism, drug stimulation, obesity, virus (hepatitis B virus and hepatitis C virus), and so on [13]. Chronic hepatitis B (CHB) is the most common pathogenic factor leading to HCC [14–16]. HBV chronic infection accounts for at least 50% of HCC patients in the worldwide [14], and even more than to 90% in some regions [17]. Transcription factors play an important role in chronic HBV infection [18]. Interestingly, studies have reported that GATA4 can regulate retrovirus infection [19–21]. However, the relationship between GATA4 and HBV is unclear.

Hepatocyte nuclear factor 4 alpha (HNF4α) is a necessary factor that regulates HBV transcriptional activity in hepatocytes. It can bind to the basic core promoter region of HBV cccDNA to promote synthesis of the 3.5 kb pgRNA [22, 23]. The continuous amplification of HBV in host cells relies on the reverse transcription process of HBV using 3.5 kb pgRNA as a template [24]. More importantly, both GATA4 and HNF4α are involved in the regulation of tissue development pathways, and GATA4 is considered to be the potential upstream of HNF4α, regulating different stages of liver development [25]. Whether there is an association between GATA4 and HNF4α in the regulation of HBV remains to be further observed.

In this study, we discovered for the first time that GATA4 may play a positive regulatory role on HBV in vitro. Meanwhile, HBV status was found to have no significant effect on GATA4. Further analysis showed that HNF4α may be located downstream of GATA4 and regulate the level of HBV transcription.

## Materials and methods

### Retrospective analysis of GATA4 expression and HBV carrying status in clinical HCC patients

19 HCC clinical patient information involved in the previous study was obtained from the Molecular Oncology and Epigenetics Laboratory of the First Affiliated Hospital of Chongqing Medical University (Chongqing, China) [12]. This study retrospectively analyzed the relationship between GATA4 expression and HBV carrier status in these patients.

### Cell lines and HBV cccDNA preparation

The normal liver cell line used in this study was L02 cells, the hepatoma or hepatoblastoma cell lines were SMMC7721, HepG2 and HepG2.2.15 cells. These cell lines were purchased from the ATCC cell bank (American Type Culture Collection, Manassas, VA, USA), and all cell lines have been tested for mycoplasma according to the manufacturer’s protocol (Solarbio, Beijing, China). The synthesis of HBV cccDNA was performed as described in a previous study [26]. Briefly, the HBV genome fragment was amplified and synthesized using the phusion high-fidelity DNA polymerase (F530L, Thermo scientific, Lithuania) and the HBV-pEASY plasmid as a template, then subjected to restriction digestion, circularization, and purification to obtain HBV cccDNA.

### Cell culture and transfection

L02 cells and SMMC7721 cells were maintained in RPMI 1640 medium (Thermo Fisher Scientific, Suzhou, China) supplemented with 10% fetal bovine serum (Biological Industries, Beit HaEmek, Israel). HepG2 cells and HepG2.2.15 cells were maintained in Dulbecco’s modified Eagle’s medium (Thermo Fisher Scientific) supplemented with 10% fetal bovine serum. All cell lines were treated with 1% penicillin and streptomycin and grown in 5% CO_2_ at 37 °C. L02 cells and SMMC7721 cells were plated and cultured overnight, then co-transfected with stable expression plasmid of GATA4 (represented as adGATA4) and HBV cccDNA for 48 h (h). HepG2 cells were plated overnight and treated with a concentration of 10 nM GATA4 small interfering RNA (siGATA4) for 24 h, and then transfected with HBV cccDNA for 48 h. HepG2.2.15 cells were only treated with siGATA4 for 48 h. Lipofectamine™ 8000 reagent (Beyotime, Shanghai, China) was used in all transfection processes according to the manufacturer’s protocol. The adGATA4 and siGATA4 are the same as in our previous study [12]. In addition, HepG2.2.15 cells were treated with a specific stimulator of HNF4α, benfluorex hydrochloride (JP-992 hydrochloride; MedChem Express, Monmouth Junction, NJ, USA) for 48 h after siGATA4 for 24 h, the concentration of benfluorex hydrochloride was the same as reported previously [27]. Each experiment was repeated independently at least three times.

### Reverse transcription polymerase chain reaction (RT-PCR) and reverse transcription quantitative PCR (RT-qPCR)

Total RNA was extracted using TRIzol reagent (Invitrogen, Carlsbad, CA, USA). Total RNA (1 µg) was reverse transcribed at 42 °C for 1 h into cDNA using GoScript™ Reverse Transcriptase (A5002, Promega, Beijing, China). HBV Genomic DNA was extracted from cells and liver tissues with a TIANamp Genomic DNA kit (TIANGEN, Beijing, Chain). All operations were performed using the manufacturers’ protocols. The mRNA levels of GATA4, HNF4α, HBV 3.5 kb pgRNA, and HBV total RNA were detected by RT-PCR or RT-qPCR, and β-actin was used as the internal control. Copies of HBV DNA from genomic DNA were determined by RT-qPCR with a serial dilution of known copies of HBV standards. The RT-qPCR was conducted using the Bio-Rad CFX connect Real-Time system with iTaq Universal SYBR® Green Supermix (Bio-Rad, Hercules, CA, USA), referring to the manufacturer’s instructions. The 2^−∆∆Ct^ method was used to calculate the Cq data of the samples obtained by RT-qPCR. At least three independent repeat experiments were performed. All primers used are listed in Table 1.

**Table.1:** Summary of primer sequences used in this study

Gene	Forward sequence (5′–3′)	Reverse sequence (5′–3′)
β-actin	GTCTTCCCCTCCATCGTG	AGGGTGAGGATGCCTCTCTT
GATA4	CCTCTACCACAAGATGAACG	CCTCTTTCCGCATTGCAAGA
HNF4α	CTAACACGATGCCCTCTCAC	GCAGGAGCTTGTAGGATTCAG
HBV 3.5 kb pgRNA	GCCTTAGAGTCTCCTGAGCA	GAGGGAGTTCTTCTTCTAGG
HBV total RNA	ACCGACCTTGAGGCATACTT	GCCTACAGCCTCCTAGTACA
HBV DNA	TTCTCCGCCTGTCGTACC	GGTTTCTGTGGGCGTTC
HDAC2	TGGACTCTTTGAGTTTTGTCAG	CATCACCATGATGAATATCTAT

### Enzyme-linked immunosorbent assays (ELISA)

The level of HBsAg and HBeAg in cell culture medium were detected by using ELISA kits according to the manufacturer’s instructions (Kehua Bioengineering, Shanghai, China). The results were judged to be effective or not in advance referencing the instructions. Subsequently, the HBsAg and HBeAg levels in each sample were calculated according to the standard curve established with HBsAg and HBeAg standard (Kangchesitan, Beijing, China).

### Western blotting analysis

Total cellular proteins were extracted using radioimmunoprecipitation assay lysis solution (strong; Beyotime), a protease/phosphatase inhibitor mix (Universal Type, 50×; Beyotime), and ultrasonic disruption. After centrifugation at 13,400 ×*g* for 15 min at 4 °C, the concentration of protein in the supernatant was detected using a bicinchoninic acid protein assay kit (Solarbio, Beijing, China). Then, 20% volume of 6× protein loading buffer (Beyotime) was added to the supernatant, all operations were followed the manufacturer's agreement. The protein was then placed at 100 °C for 5–8 min for denaturation. Subsequently, the protein samples were electrophoretically separated on a 7.5% sodium dodecyl sulfate polyacrylamide gel, then transferred onto a 0.45 μm polyvinylidene fluoride membrane (Merck KGaA, Darmstadt, Germany) in an ice bath. The membrane was blocked with QuickBlock™ Western Blocking Solution (Beyotime) for 15 min, washed three times with tris-buffered saline containing 0.1% Tween 20 for 15 min each time, and the incubated with the primary antibody at 4 °C overnight. The primary antibodies used were as follows: rabbit anti-HNF4α antibody (bs-3828R, BIOSS, Beijing, China; 1:1000), mouse anti-human GATA4 monoclonal antibody (sc-25310, Santa Cruz Biotechnology, Shanghai, China; 1:1000), and mouse anti-tubulin antibody (sc-5274, Santa Cruz Biotechnology, Shanghai, China; 1:1000) as the internal control. After incubation with the primary antibodies, the membrane was washed with tris-buffered saline containing 0.1% Tween 20 and incubated with the anti-rabbit or anti-mouse secondary antibody for 1 h. Proteins were visualized using an ultra-high sensitivity enhanced chemiluminescence kit (HY-K1005, MedChem Express). All assays were performed three times independently.

### Immunofluorescence analysis

Cells were seeded into 24-well plates with microcoverslips overnight and then transfected with a GATA4 plasmid and cultured for 48 h. Cells were fixed with 4% paraformaldehyde for 30 min, treated with rupture working solution (PN00014) for 10 min, and blocked with serum for 30 min. And then the slides were incubated with primary antibodies at 4 °C overnight. The primary antibodies used were as follows: GATA4 mouse monoclonal antibody, Clone OTI9F9 (TA500091S, OriGene, Beijing, China; 1:100), HNF4α (C11F12) Rabbit monoclonal antibody (3113S, Cell Signaling Technology, Boston, USA, 1:1500). Subsequently, cells were incubated with Alexa Fluor CY3-conjugated or 488-conjugated goat antimouse or goat anti-rabbit secondary antibodies (Jackson ImmunoResearch, West Grove, PA, USA) for 1 h at 37 C in the dark. Finally, the nuclei were counterstained with 4^0^-6-diamidino-2-phenylindole (DAPI, C0060, Solarbio, Beijing, China) in the dark for 10 min. Micrographs were captured by using fluorescence microscopy. Assays were performed in three separate times.

### Statistical analysis

Data were analyzed using SPSS 22.0 software (IBM, Chicago, IL, USA) and ImageJ v1.8.0 (NIH, Bethesda, Maryland, USA). Continuous variables are reported as means ± standard deviations calculated by Prism Software version 5.0 (GraphPad Software, La Jolla, CA, USA). Data with a normal distribution were compared with a two-tailed Student’s t-test. All experiments were repeated independently at least three times. Significance was set at *P* < 0.05 (non-significance at *P* ≥ 0.05).

## Results

### Expression of GATA4 in cells

**Fig. 1:**
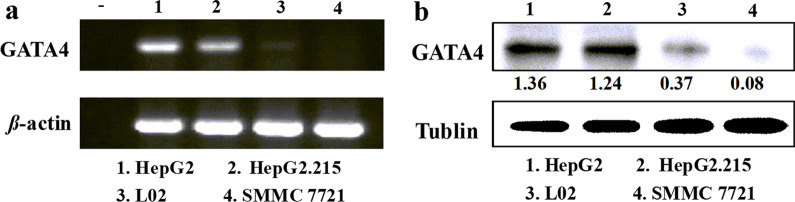
The expression of GATA4 in hepatocyte lines. **a** GATA4 mRNA level in cells detected via reverse-transcription PCR (RT-PCR). **b** Protein of GATA4 (51 kDa) analyzed by western blotting (WB), and tubulin (55 kDa) was used as a loading control

We first verified the expression of GATA4 in different normal liver cell lines or HCC cell lines via RT-PCR and western blotting (WB) to determine the appropriate cell models. The mRNA levels (Fig. 1a) and protein levels (Fig. 1b) results of these cells showed that GATA4 was expressed at low levels in the L02 cells and SMMC7721 cells, but at highly levels in the HepG2 cells and HepG2.2.15 cells. The expression level of GATA4 in different cells was the basis for the selection of cell models. Therefore, in this study, plasmid adGATA4 was treated in L02 cells and SMMC7721 cells, and siGATA4 was interfered in HepG2 cells and HepG2.2.15 cells.

### GATA4 overexpression enhances the levels of HBV DNA, HBsAg and HBeAg, 3.5 kb pgRNA and total RNA

**Fig. 2:**
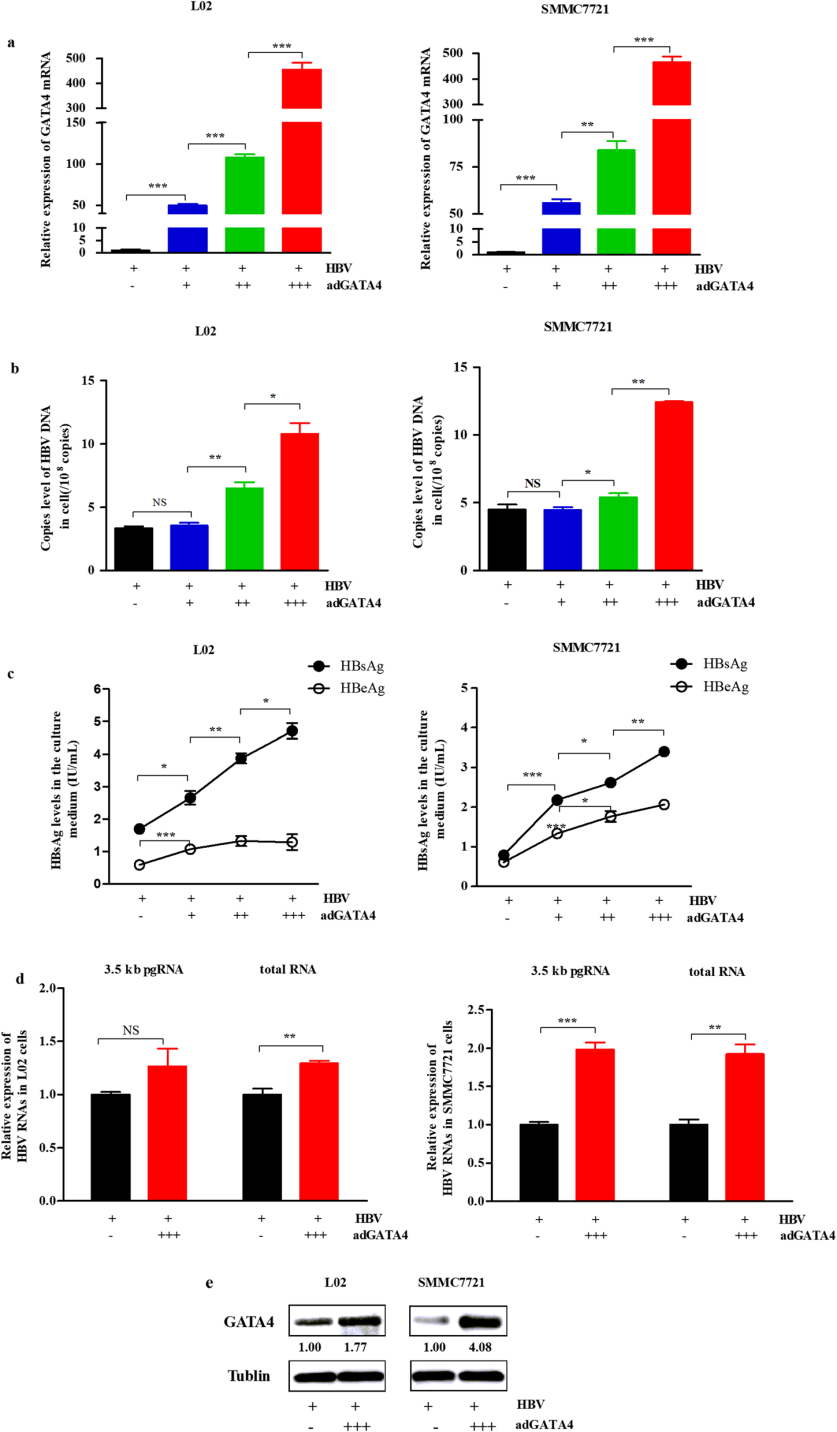
Overexpression of GATA4 may promote HBV in L02 cells and SMMC7721 cells, respectively. L02 cells and SMMC7721 cells were transfected HBV cccDNA (500 ng per 1 mL 1640 medium, as 500 ng/mL) and adGATA4 for 48 h. (Description for the final concentration of adGATA4: − , control; + , 100 ng/mL; ++ , 200 ng/mL; +++ , 400 ng/mL.) **a** Relative expression of GATA4 mRNA in cells detected via RT-qPCR, β-actin was used as the internal control. **b** Analysis of HBV DNA copies in cells determined by qPCR. **c** HBsAg and HBeAg levels in the culture medium detected through enzyme-linked immunosorbent assays. **d** Relative levels of HBV 3.5 kb pgRNA and total RNA in cells analyzed via RT-qPCR, β-actin was used as the internal control. **e** Protein of GATA4 (51 kDa) analyzed via WB. All statistics are marked as NS *P* > 0.05, *P < 0.05, **P < 0.01, ***P < 0.001

To verify whether GATA4 has a regulatory effect on HBV, adGATA4 with different mass concentrations and equal amounts of HBV cccDNA were co-transfected into L02 cells and SMMC7721 cells, respectively. The results showed that the expression of GATA4 increased with the increasing adGATA4 concentrations (all *P* < 0.01, Fig. 2a). The levels of HBV DNA copies in the cells increased significantly in the treatment group with high concentration of adGATA4 (Fig. 2b). The levels of HBsAg and HBeAg secreted into the culture medium were consistent with GATA4 expression, the increase of HBsAg was particularly significant (all *P* < 0.05, Fig. 2c).

Then, the levels of 3.5 kb pgRNA and total RNA of HBV were further observed after transfection with adGATA4 with a final concentration of 400 ng/mL. The results showed that the levels of 3.5 kb pgRNA and total RNA of HBV were increased compared with the normal group (Fig. 2d), although the increase in the level of HBV 3.5 kb pgRNA in L02 cells was not significant (*P* > 0.05). GATA4 protein was increased after transfection with adGATA4 (Fig. 2e). General results revealed that GATA4 may be involved in promoting the expression and replication of HBV.

### siGATA4 negatively regulates HBV

**Fig. 3:**
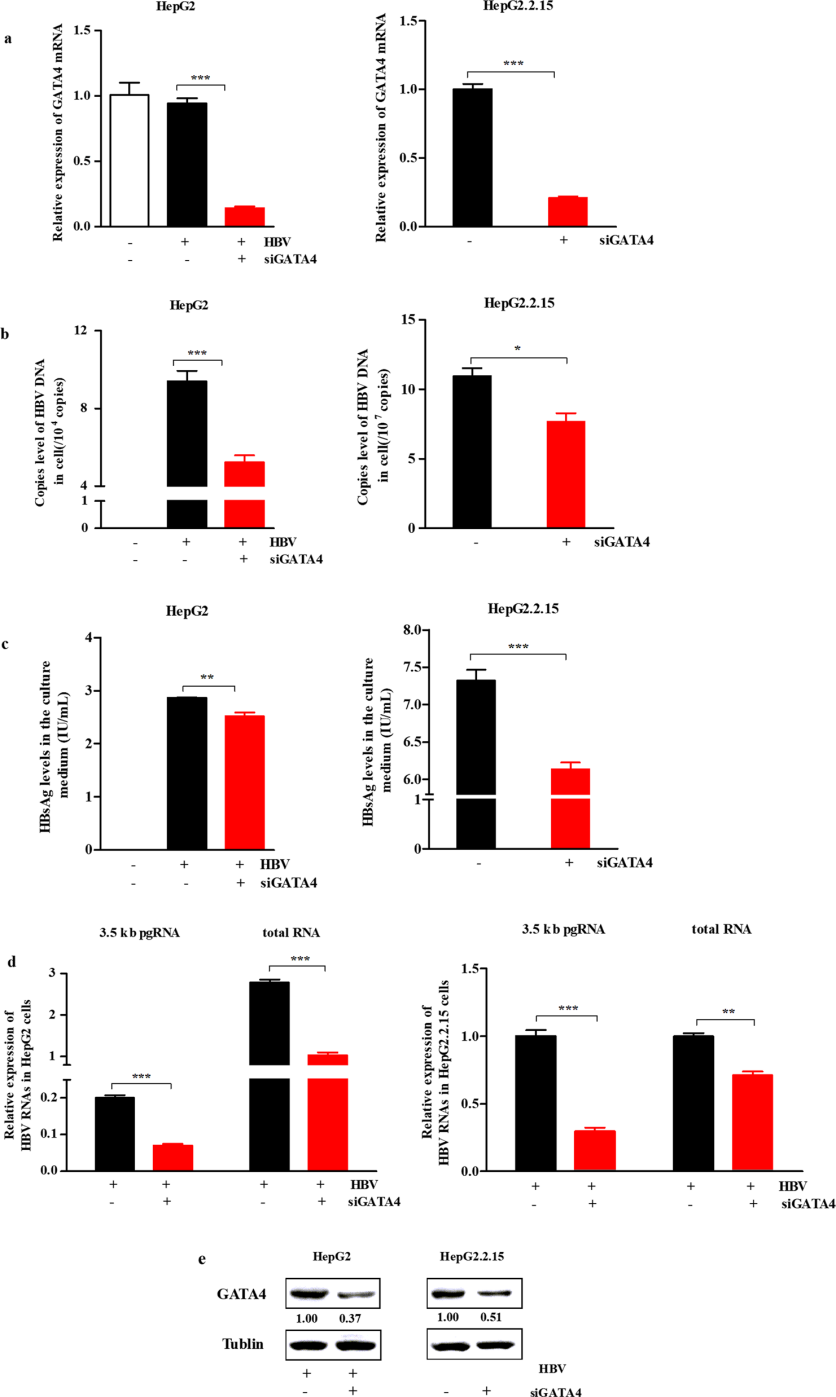
siGATA4 negatively regulates HBV. HepG2 cells were transfected with siGATA4 (10 nM) for 24 h, and then transfected with HBV cccDNA (500 ng/mL) for 48 h. HepG2.2.15 cells carry the complete genome of HBV, so these were only transfected with siGATA4 for 48 h. **a** Relative mRNA of GATA4 in cells detected via RT-qPCR, β-actin was used as the internal control. **b** Analysis of HBV DNA copies in cells determined by qPCR. **c** HBsAg levels in the culture medium detected through enzyme-linked immunosorbent assays. **d** Relative levels of HBV 3.5 kb pgRNA and total RNA in cells analyzed via RT-qPCR, β-actin was used as the internal control. **e** Protein of GATA4 (51 kDa) analyzed via WB. All statistics are marked as NS *P* > 0.05, *P < 0.05, **P < 0.01, ***P < 0.001

To further verify the effect of GATA4 on HBV, we silenced GATA4 expression in HepG2 cells and HepG2.2.15 cells using siGATA4. The expression of GATA4 was effectively suppressed in the siGATA4 group (all *P* < 0.001, Fig. 3a). The levels of HBV DNA and HBsAg were reduced in cells (Fig. 3b, c). Moreover, the levels of 3.5 kb pgRNA and total RNA of HBV were significantly decreased after siGATA4 (all *P* < 0.01, Fig. 3d). GATA4 protein was also decreased after transfection with siGATA4 (Fig. 3e). The above results converse to prove the promoting effect of GATA4 on HBV.

### GATA4 may be an upstream transcription factor regulating HBV

**Fig. 4:**
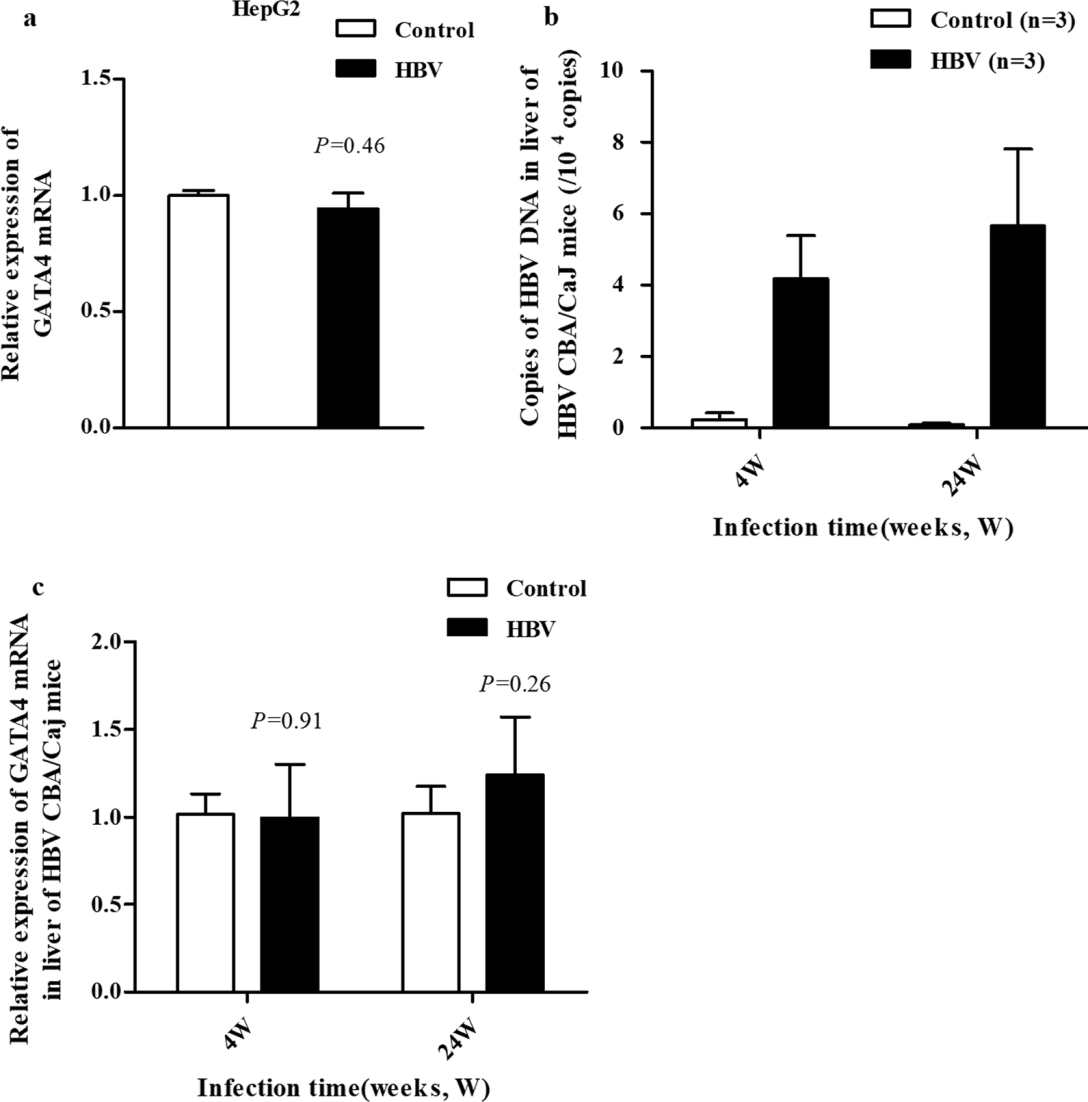
HBV had no significant effect on GATA4 in HBV cccDNA cell model and mouse model. HepG2 cells were transfected with HBV cccDNA (500 ng/mL) for 48 h. **a** Relative mRNA of GATA4 in cells detected via RT-qPCR, β-actin was used as the internal control. *P* = 0.46 vs. control group. **b** Analysis of HBV DNA copies in liver of HBV CBA/CaJ mouse models determined by qPCR, the average copy numbers of HBV DNA are 4.2 × 10^4^ copies and 5.7 × 10^4^ copies at 4 weeks and 24 weeks, respectively. **c** Relative mRNA of GATA4 in liver of HBV CBA/CaJ mouse models (n = 3) detected via RT-qPCR, β-actin was used as the internal control. *P* = 0.91 or *P* = 0.26 vs. control group

In order to observe whether HBV affected the expression of GATA4, we used HBV cccDNA cell model to observe the mRNA level of GATA4, and the results showed there was no clear difference in GATA4 expression in HepG2 cells before and after transfection with HBV (*P* = 0.46, Fig. 4a). Subsequently, the copies of HBV DNA and the mRNA level of GATA4 were analyzed in liver tissue samples of the HBV mouse models at 4 and 24 weeks [26]. The results showed that the average copy numbers of HBV DNA are 4.2 × 10^4^ copies and 5.7 × 10^4^ copies in the liver tissue of the HBV CBA/CaJ mice (Fig. 4b). The expression of GATA4 did not significantly change in HBV models group compared with the control mice (*P* = 0.91 or *P* = 0.26, Fig. 4c). The results demonstrated that HBV had no significant effect on GATA4 in HBV cccDNA cell models and mouse models.

### GATA4 reduced expression in HCC patients regardless of the HBV and HBsAg status of patients

The levels of GATA4 in clinical patients (n = 19) were based on the results of the previous study [12]. The results showed that 68.4% of the patients were HBV and HBsAg positive, and 31.6% were HBV and HBsAg negative in the 19 HCC patients. Compared with adjacent tissues, the high or low expression of GATA4 in cancer tissues accounted for 23.1% and 76.9%, 16.7% and 83.3% in HBV-HCC patients and in non-HBV-HCC patients, respectively. The results showed that regardless of the patient's HBV and HBsAg status, the expression level of GATA4 decreased in liver cancer tissues of most patients (Table 2).

**Table.2:** Retrospective analysis of GATA4 expression and HBV carrying status in clinical HCC patients

HBV and HBsAg in HCC patients	Positive 68.4% (13/19)		Negative 31.6% (6/19)	
GATA4 expression	High	Low	High	Low
Total number	3	10	1	5
Ratio	23.1% (3/13)	76.9% (10/13)	16.7% (1/6)	83.3% (5/6)

### GATA4 may be associated with the host factor HNF4α that regulates HBV transcription

**Fig. 5:**
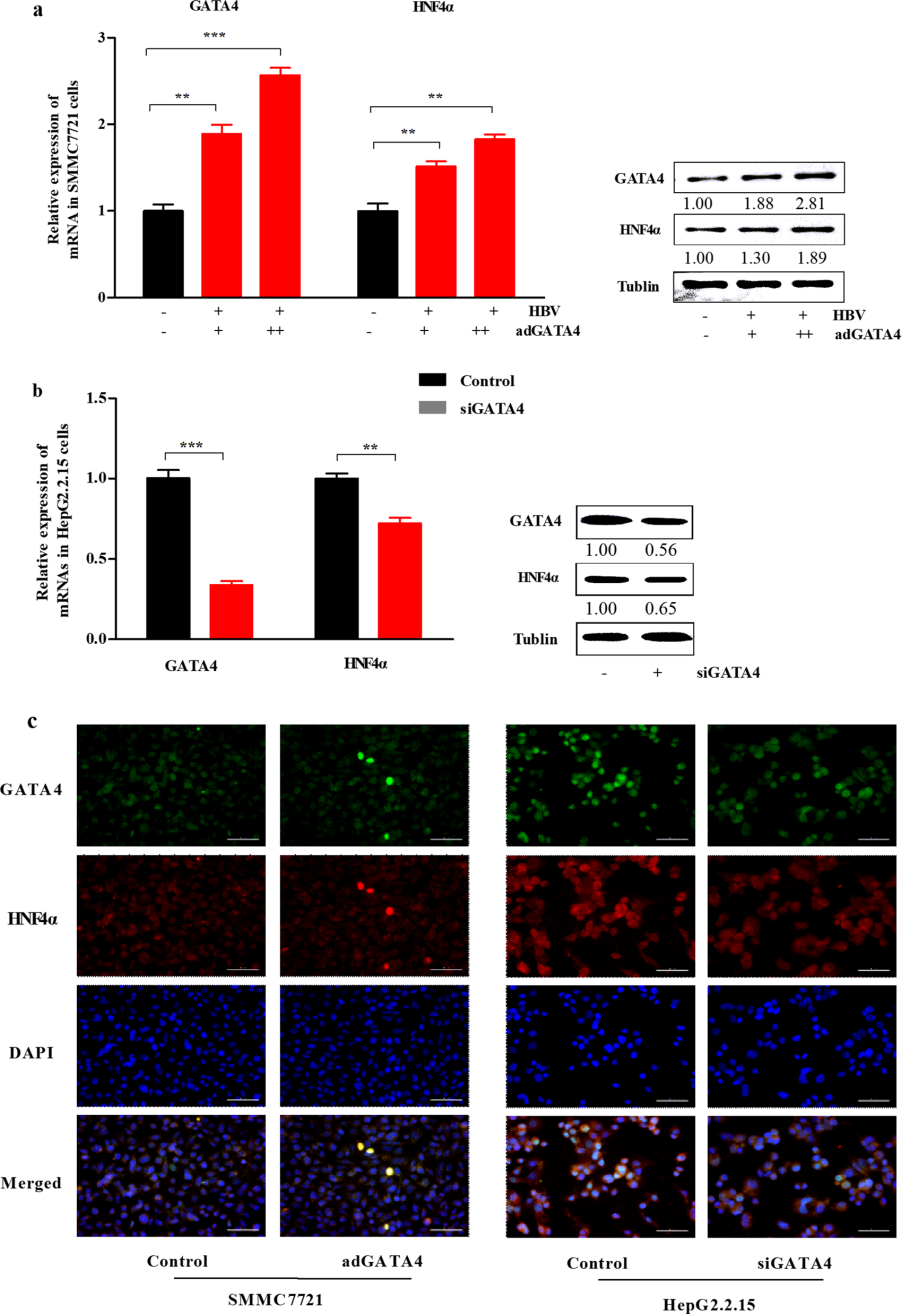
The relationship between GATA4 and HNF4α in cells. SMMC7721 cells and HepG2.2.15 cells were transfected with adGATA4 or siGATA4 for 48 h. **a**, **b** Relative mRNA and protein of HNF4α in cells measured by RT-qPCR and WB. Protein of GATA4 (51 kDa) and HNF4α (52 kDa) were analyzed by WB, and tubulin (55 kDa) was used as a loading control. All statistics are marked as NS *P* > 0.05, *P < 0.05, **P < 0.01, ***P < 0.001. **c** Immunofluorescence was used to evaluate the relationship between GATA4 and HNF4α

To observe whether HNF4α is related to the regulation of HBV by GATA4, HNF4α was detected in adGATA4 or siGATA4 cell models using RT-qPCR or western blotting (WB) assay. The results suggested that the mRNA and protein levels of HNF4α were concomitant with the expression of GATA4 (Fig. 5a, b). The results of immunofluorescence analysis of GATA4 and HNF4α in cells showed that, compared with the control group, the expression of HNF4α was increased or decreased in the cells transfected with adGATA4 or siGATA4 (Fig. 5c). Collectively, these results indicate that GATA4 may be associated with the host factor HNF4α.

### Stimulating HNF4α alleviates the inhibitory effect of siGATA4 on HBV in HepG2.2.15 cells

**Fig. 6:**
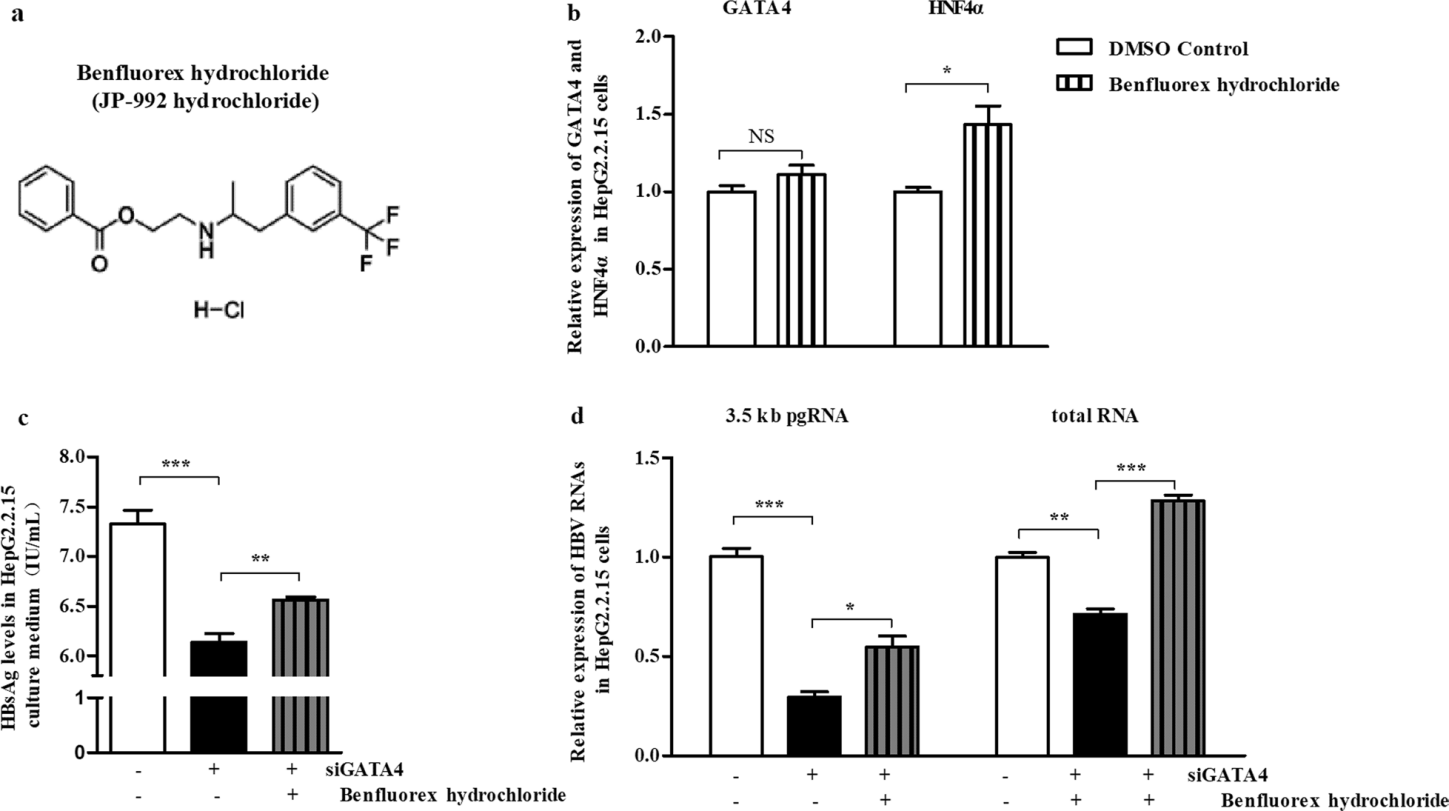
Stimulating HNF4α alleviates the inhibitory effect of siGATA4 on HBV in HepG2.2.15 cells. HepG2.2.15 cells were treated with benfluorex hydrochloride (Final concentration was 1 nM), a specific stimulator of HNF4α, for 48 h after siGATA4 for 24 h. **a** Chemical structure of the HNF4α specific stimulator, benfluorex hydrochloride (JP-992 hydrochloride). **b** Relative mRNA of GATA4 detected by RT-qPCR. NS, *P* > 0.05 vs. control of siGATA4. **c** HBsAg levels in the culture medium measured via ELISA kit assay. **d** Relative levels of HBV 3.5 kb pgRNA and total RNA detected via RT-qPCR, β-actin was used as the internal control

**Fig. 7:**
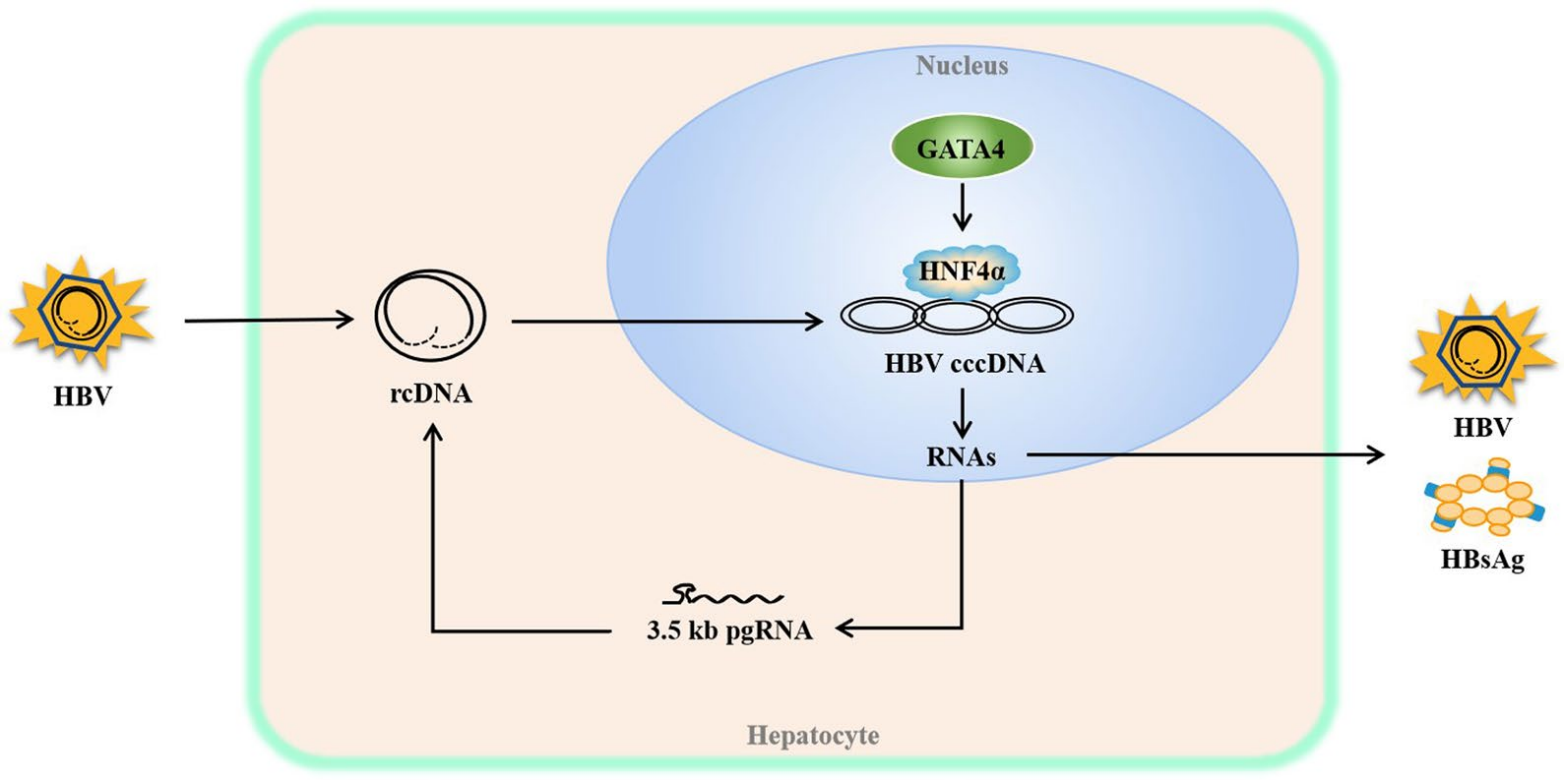
A possible pathway of GATA4 regulation HBV, GATA4 may promote HBV activity through up-regulating HNF4α

To further confirm the role of HNF4α in HBV regulation by GATA4, GATA4 and HNF4α were observed in HepG2.2.15 cells. The results displayed that HNF4α levels could be specifically upregulated by benfluorex hydrochloride but had no significant effect on GATA4 in HepG2.2.15 cells (Fig. 6a, b). Meanwhile, HBsAg and HBV RNAs were significantly reversed by benfluorex hydrochloride in siGATA4 HepG2.2.15 cells (Fig. 6c, d). The upregulation of HNF4α attenuated the inhibitory effect of siGATA4 on HBV. Taken together, the overall results revealed that GATA4 may play a role in the upstream of HNF4α in HBV-related pathways (Fig. 7).

## Discussion

HBV is one of the smallest hepatotropic DNA viruses among all identified to date, and HBV infection is one of the most difficult to be eliminated completely [28, 29]. The possibility of HBV being reactivated is still extremely high after receiving anti-tumor treatment, even in HCC patients whose HBV has turned negative [30–32]. HBV transcription, which uses HBV covalently closed circular DNA (cccDNA) as a template to synthesize 3.5 kb pgRNA and multiple messenger RNAs, is an important process for HBV persistent infection [33, 34]. This process depends on the activities of various transcription factors and chromatin-modifying enzymes [35, 36]. Therefore, exploring the effect of GATA4 on HBV has significant research value for the treatment of HBV-HCC.

The synthesis of various antigens in HBV utilizes different messenger RNA as the template, among which the HBsAg is the main serum marker of HBV [37]. The levels of HBsAg and HBV DNA are important indicators for judging the active status of HBV replication [38]. 3.5 kb pgRNA is the template for reverse transcription synthesis of HBV genome relaxes circular DNA (rcDNA), so it is a key intermediate to ensure HBV continuous replication [34]. The most important finding of this study was that GATA4 had a positive regulatory effect on HBV. GATA4 overexpression promoted the secretion of HBsAg, increased the copies of HBV DNA, and enhanced the synthesis of HBV 3.5 kb pgRNA and total RNA. In contrast, the levels of HBsAg, HBV DNA and HBV RNAs were downregulated by siGATA4. Although the present study found that GATA4 could promote HBV, it has not yet been verified by southern blotting or northern blotting.

HNF4α is widely regarded as one of the key factors in the activation of HBV cccDNA and the synthesis of pgRNA [23, 39–41]. Previous studies found that HNF4α binds to the HBV genome enhancer, IEnhI/Xp [23, 42]. Moreover, HNF4α participates in the formation of cccDNA chromatin microsomes by binding to the core promoter group region of cccDNA, initiating HBV replication and transcription [22, 43]. HNF4α is also a potential target for the treatment of HBV [44–48]. In this study, the results demonstrated that HNF4α was increased simultaneously after the up-regulation of GATA4. In contrast, up-regulation of HNF4α could only increase HBV markers, but had no effect on GATA4. These results suggest that GATA4 may be a potential upstream regulator of HNF4α, and a stimulus of HBV replication and transcription. Remarkably, the direct interaction between GATA4 and HNF4α in this study had not be identified using a luciferase reporter assay and an immunoprecipitation assay. Therefore, the underlying mechanism requires further investigation.

## Conclusion

Previous studies have found that GATA4 is a potential anti-HCC therapeutic target, the current study found that the expression of GATA4 was also decreased in cancer tissues of the most HBV-HCC patients. Furthermore, we innovatively found that GATA4 can promote HBV replication and transcription, and the regulation effect of GATA4 on HBV may be related to HNF4α. The overall results imply that the combination of anti-HBV agents should be considered in HBV-HCC patients when GATA4 is used as the target of antitumor gene therapy.

## Data Availability

All data generated or analyzed during this study are included in this published article.

## Abbreviations

HBV: Hepatitis B virus
HBsAg: Hepatitis B surface antigen
HBV DNA: Hepatitis B virus DNA
cccDNA: Covalently closed circular DNA
pgRNA: Pre-genomic RNA
HBV RNA: Hepatitis B virus RNA
HCC: Hepatocellular carcinoma
GATA4: GATA binding protein 4
HNF4α: Hepatocyte nuclear factor 4 alpha
adGATA4: Expression plasmid of GATA4
siGATA4: GATA4 small interfering RNA
